# Headaches, Their Causes and Their Cure

**Published:** 1856-07

**Authors:** 


ART. X.—*Headaches, their Causes and their Cure.* By HENRY G. WRIGHT, M. D., M. R. C. S. L., Fellow Royal Med. Chir. Soc., Physician to the St. Pancras Royal Dispensary. New York: S. S. & WM. WOOD. 1856.

This is a sensible little duodecimo. The taking up of a single symptom, like headache, and tracing out its various causes and connections is always a useful task, and though there is no great evidence of personal observation or profound learning in Dr. Wright's little book, it will still be useful to the professional reader.

The recommendations of treatment are mostly appropriate, and the important distinction between the headaches of different ages, and their symptomatic importance, is carefully maintained throughout.

